# A quaternary equation for interdisciplinary medical research (IMR)

**DOI:** 10.1186/1479-5876-10-S2-A49

**Published:** 2012-10-17

**Authors:** Marc Shuman, Jim Wells, K Shokat, S Bernales, P Walter, Leonard Shultz, Neal Goodwin

**Affiliations:** 1 Departments of Medicine, Univ. of California Schools of Medicine & Pharmacy, San Francisco CA, USA; 2 Departments of Pharmaceutical Chemistry, Univ. of California Schools of Medicine & Pharmacy, San Francisco CA, USA; 3 Department of Cellular and Molecular Pharmacology, Univ. of California Schools of Medicine & Pharmacy, San Francisco CA, USA; 4 Departments of Biochemistry & Biophysics, Univ. of California Schools of Medicine & Pharmacy, San Francisco CA, USA; 5 The Jackson Laboratory, Sacramento CA, USA

## 

A popular model for developmental therapeutics is one in which drugs are developed by biotechnology and pharma. Candidate molecules are then handed off to academia and Clinical Research Organizations (CRO’s) for clinical testing. We have embarked on a different approach whereby 1. Potential targets are identified in a candidate pathway, 2. Gene expression/genomics are interrogated in normal and diseased human tissues for their relevance, 3. Highly specific chemical reagents against putative targets are developed to determine their importance, 4. Model compounds are tested in pre-clinical cell and mouse models, 5. Collaborations are established with biotechnology/pharma for drug development. We have taken this approach with the goal of stimulating drug development in an as yet relatively unexplored important survival pathway, the Unfolded Protein Response. Interest in this complex pathway, expressed in all eukaryotic organisms, has recently surfaced, and particularly in cancer and neurodegenerative diseases. Our team includes faculty with expertise in high throughput drug screening, kinase chemistry, cell biology, and clinical research. Specific compounds have been identified, further modified and tested against all 3 branches of the UPR: Ire1, PERK and ATF6 using chemical and cell – based assays. In addition, novel transgenic animals have been established in which there is the exciting potential for recreating the orphan disease, Multiple Myeloma. Using primary bone marrow from patients with this disease, we hope to be able to screen drugs, in vivo, that will predict individual patients’ response to treatment.

